# Review of Lutetium-177-labeled Anti-prostate-specific Membrane Antigen Monoclonal Antibody J591 for the Treatment of Metastatic Castration-resistant Prostate Cancer

**DOI:** 10.7759/cureus.7107

**Published:** 2020-02-26

**Authors:** Muhammad O Niaz, Michael Sun, Marigdalia K Ramirez-Fort, Muhammad J Niaz

**Affiliations:** 1 Internal Medicine, Sharif Medical City Hospital, Lahore, PAK; 2 Internal Medicine, Weill Cornell Medicine, New York, USA; 3 Life Sciences, Biofort Corp., Guaynabo, PRI; 4 Urology, Weill Cornell Medicine, New York, USA; 5 Physiology / Pathology, San Juan Bautista School of Medicine, Caguas, PRI

**Keywords:** prostate cancer, radioimmunotherapy, monoclonal antibody

## Abstract

Prostate cancer is the most common non-cutaneous cancer in men in the United States and is the second most common cause of cancer deaths after lung cancer in men. Despite all advances in the field of prostate cancer imaging and treatment, currently, it is sub-optimally responsive to all available treatment options. Radioimmunotherapy with a monoclonal antibody (mAb), J591, has shown promising results in the treatment of prostate cancer. J591 is a deimmunized mAb that targets the extracellular domain of prostate-specific membrane antigen (PSMA), a surface-bound and internalizing glycoprotein that is upregulated in prostate cancer. Phase I/II clinical trials have shown accurate tumor targeting, biochemical and radiographic responses, and increased overall survival in patients with mCRPC with tolerable, predictable, and reversible myelotoxicity. Ongoing studies focus on improving the therapeutic index of radiolabeled J591. Herein, the literature on published clinical trials involving therapeutic J591 conjugated to b-emitter, lutetium-177 for mCRPC, is sequentially reviewed.

## Introduction and background

Prostate cancer is one of the most commonly found non-cutaneous tumors in men in the United States, with an estimated 174,650 new cases and 31,620 deaths in the year 2019 [1]. Treatment options include surgery, radiation, conservative management (active surveillance or watchful waiting), and androgen deprivation therapy [2-3]. Although these options can be effective for localized tumors, some patients ultimately develop castration-resistant prostate cancer [4]. Currently, the most recognizable target for the management of this castration-resistant prostate cancer (mCRPC) is prostate-specific membrane antigen (PSMA). PSMA is a transmembrane protein overexpressed by prostate cancer cells, although found on other tissues (e.g., kidney, salivary glands, and intestines) too [5]. Many molecules have been developed that target this antigen, including J591. It is a monoclonal antibody that binds to PSMA and has the property of internalization once bound to PSMA [6-7]. Various phase I and phase II trials have been conducted using 177Lu-J591, demonstrating promising results with predictable, dose-dependent, and reversible hematological toxicities. Herein, we summarize the published ^177^Lu-J591 clinical data for the treatment of mCRPC. Only trials with full results published in the form of original articles were included in this review. Table 1 shows the salient characteristics of all Lu-J591 trails discussed in this review.

**Table 1:** Summary of all 177Lu-J591 trials PSA: prostate-specific antigen

Study Name	First author	Year	Patients (n)	^177^Lu-J591 Dose, mCi/m^2^	Dosing schedule	Any PSA response n (%)
Phase I single-dose escalation	Bander et al.	2005	35	10 – 75	Single	20/35 (57%)
Phase II single dose	Tagawa et al.	2013	47	65 – 70	Single	28/47 (60%)
Phase I/II dose fractionation	Tagawa et al.	2019	49	20 - 45 40 – 45	Two doses, given two weeks apart	27/49 (55.1%)
Hyperfractionated pilot study	Niaz et al.	2020	6	25	Biweekly until the emergence of > grade 2 toxicity	2/6 (33.3%)

## Review

Phase I dose-escalation study with ^177^Lu-J591

A phase I dose-escalation study with ^177^Lu-J591 was the first trial to use radiolabeled ^177^Lu-J591 clinically [8]. The primary objectives of this trial were to determine the maximum tolerated dose (MTD), toxicity, human anti-J591 response, pharmacokinetics, organ dosimetry, targeting, and biologic activity of ^177^Lu-J591 in patients with androgen-independent prostate cancer. The secondary objective was to assess the anti-tumor response. Evaluation Program Common Toxicity Criteria (version 2.0) was utilized. Dose-limiting toxicity (DLT) was defined as grade 4 thrombocytopenia (platelet count less than 10x10^9^/L) and/or grade 4 neutropenia (absolute neutrophil count (ANC) < 0.5 x 10^9^) for greater than five days, and other toxicities consisting of any grade 3 or more non-hematologic toxicity that could be attributable to ^177^Lu-J591. The development of grade 2 toxicity or signs of an allergic infusion reaction to^ 177^Lu-J591 would preclude further treatment with ^177^Lu-J591. Patients with a previous histologic diagnosis of prostate cancer with a rising prostate-specific antigen (PSA) level and/or tumor progression on radiologic studies (including bone scan, computed tomography (CT), and/or magnetic resonance imaging (MRI)), despite androgen deprivation therapy (ADT) and castrate testosterone levels were eligible for the study. No pre-selection based on PSMA expression was made.

A total of 35 subjects received treatment, of whom 16 received up to three doses. The median baseline PSA was 29.6 ng/mL (range 2.3-2746.0). Ninety-seven percent (97%; n=34) had been previously treated with hormonal therapy and 37% (n=10) with chemotherapy. A total of 10 mg/m^2^ of humanized J591 mAb conjugated to escalating doses of ^177^Lu (ranging from 10 mCi/m2-75 mCi/m2) were administered per patient. Accurate tumor targeting was confirmed with radiographic imaging in 30 patients. Myelosuppression was dose-limiting at 75 mCi/m2. Therefore, 70-mCi/m2 ^177^Lu-J591 was determined to be the MTD. Repeat dosing at 45 to 60 mCi/m2 also resulted in dose-limiting myelosuppression; up to three doses of 30 mCi/m2 could be safely administered. More than grade 1 non-hematologic toxicity was rare.

While the majority of patients received sub-therapeutic doses in this dose-escalation phase I study, four (11.4%) patients experienced more than a 50% PSA decline that lasted three to eight months. Additionally, 16 (46 %) subjects experienced PSA stabilization for a median of 60 days (1 - 21+ months). No human anti-J591 antibody response was detected. Hence, the phase I trial demonstrated acceptable toxicity with excellent targeting of known sites of prostate cancer metastasis and anti-tumor response that warranted further investigation.

Phase II single-dose study with ^177^Lu-J591

After establishing the safety and maximum tolerated dose (MTD),^ 177^Lu-J591 investigation progressed into a dual center, phase II clinical trial [9]. The primary endpoint of the study was PSA response and/or measurable disease response; the secondary endpoint was toxicity assessment. Patients with histologically confirmed prostate cancer were eligible. The study consisted of two cohorts of patients with mCRPC. The first cohort included 15 patients that received 65mCi/m2 ^177^Lu-J591 and the second cohort included 17 patients who received 70mCi/m2 ^177^Lu-J591. An expansion cohort of 15 patients received 70 mCi/m2 to verify the response rate and examine biomarkers. A total of 47 patients were treated in this study. Planar/single-photon emission computerized tomography (SPECT) scans were performed six to eight days after ^177^Lu-J591 infusion. Radiographic images were assigned visual scores ranging from 0-4, using the following criteria: 0 (no uptake), 1 (weakly positive), 2 (definitely positive), 3 (equal intensity to the liver), 4 (greater uptake than liver).

Overall, 28 patients (59.6%) experienced a PSA decline with 17 patients (36.2%) experiencing more than 30% decline in PSA following a single dose of ^177^Lu-J591. Twelve (25.53 %) patients had a measurable disease as defined by the response evaluation criteria in solid tumors (RECIST) criteria [10]. Of these 12 patients, one (8.3%) demonstrated a radiographic partial response while eight (66.6%) showed stable disease. By a semi-quantitative analysis of images, PSMA planar/SPECT successfully detected sites of metastatic disease in most patients (n=44, 93.6%). A poor visual score on the above described semi-quantitative scale was associated with a lower PSA response.

Myelosuppression was the most prominent toxicity with 22 (47%) patients developing grade 4 thrombocytopenia without hemorrhage with a nadir at four weeks. Nearly all patients (97.9%) demonstrated a complete recovery in platelet count; 30% of patients required platelet transfusions (median 2 (1-4)). Twelve (12; 25.5%) patients developed grade 4 neutropenia with a median duration of five days (range 2-17 days); one patient developed a single episode of febrile neutropenia. Nine (19.1%) patients received filgrastim or pegfilgrastim. Overall, ^177^Lu-J591 was well-tolerated with reversible myelosuppression.

Phase I/II fractionated dose study with ^177^Lu-J591

Following the above-mentioned phase I and II trials of^ 177^Lu-J591, efforts were undertaken to optimize the therapeutic response and to minimize toxicity [11]. Dose fractionation of ^177^Lu-J591 was proposed in an effort to deliver a greater cumulative dose of ^177^Lu with less toxicity. A phase I/II dose-fractionation trial was conducted. The primary endpoints were: determination of the maximum tolerated dose (MTD), dose-limiting toxicity (DLT), and recommended phase II dose (RP2D). Secondary endpoints included determinations of proportional PSA decrease, overall survival (OS), and treatment‐emergent adverse events.

Adult men with mCRPC were eligible. Twenty-eight (28) patients were treated with two doses of ^177^Lu-J591, two weeks apart, in six cohorts ranging from 20 to 45 mCi/m2 (0.74‐1.67 GBq/m2) per dose. No dose-limiting toxicity (DLT) was observed in cohorts 1‐5 (up to 40 mCi/m2 (1.48 GBq/m2) ×2). In the fifth cohort, where patients received two doses of 40 mCi/m2 (1.48 GBq/m2), one patient withdrew from the study after developing a grade 2 infusion reaction following the first dose. In cohort 6, where patients received two doses of 45mCi/m2 (1.67 GBq/m2), two subjects experienced grade 4 neutropenia lasting more than seven days (without fever). Cohorts 5 and 6 receiving 40mCi/m2 and 45 mCi/m2 doses, respectively, were determined to be the recommended phase 2 doses (RP2Ds). Both cohorts were subsequently expanded to 16 and 17 patients, respectively. A total of 49 patients were treated on this trial. Planar/SPECT imaging was obtained six to eight days after ^177^Lu‐J591 infusion and images were scored for semi-quantitative PSMA expression using the same criteria as described in the above phase II study.

Overall, 27 (55.1%) patients showed a PSA response with 16 (32.7%) patients showing more than a 30% PSA response. In cohort 6 (45mCi/m2), 15 (88%) patients had some PSA decrease while 10 (58.8%) had a more than 30% response and five (29.4%) had more than a 50% PSA response. Thirty-nine (39; 79.6%) patients had ^177^Lu‐J591 uptake on planar imaging in known sites of disease when compared with baseline CT and/or MRI and bone scan images. Based on visual scoring, a higher PSMA expression correlated with a more favorable PSA response. The overall survival (OS) for all cohorts was 23.6 months (95% CI, 15.0‐32.2). Patients in cohort 6 demonstrated an OS benefit of 42.3 months (range, 19.9‐64.7). As expected, myelotoxicity was the DLT, with a majority of patients experiencing hematologic toxicity. Overall, 27 (55.1%) patients developed all grade anemia, 40 (81.6%) developed all grade neutropenia, and 46 (93.9%) developed all grade thrombocytopenia. Nineteen (19; 38.8%) patients developed grade 4 thrombocytopenia with 14 (28.5%) patients requiring platelet transfusions (median number of transfusions = two (range 1 - 7)). Fourteen of these 19 patients with grade four thrombocytopenia have had complete recovery within a median of 26 days while three patients experienced recovery to grade one, all with a concurrent progressive disease by PSA. Subsequent bone marrow biopsies in these patients showed the bone marrow infiltration of the disease without any myelodysplastic changes. The important conclusion of this trial is that fractionation of ^177^Lu-J591 did allow for the administration of a greater cumulative dose (and, presumably, absorbed radiation dose) and resulted in improved PSA response and OS. This dose fractionation concept is also being studied with other radiolabeled therapies for prostate cancer, e.g. ^177^Lu-PSMA-617, with favorable results [12].

Pilot study of the hyperfractionated dosing of ^177^Lu-J591

After phase I and II single-dose studies of ^177^Lu-J591 demonstrated safety and efficacy with dose-response, and dose fractionation of ^177^Lu-J591 permitted higher cumulative dose administration with less myelosuppression per similar cumulative dose in single-dose studies, the idea of the hyperfractionation of ^177^Lu-J591 emerged [8-9,11-13]. The researchers hypothesized that the hyperfractionation of ^177^Lu-J591 will allow the safer administration of a higher cumulative dose with less potential toxicity.

Six men (median age of 68.6 years) with a histologically confirmed progressive mCRPC were enrolled. ^177^Lu-J591 was administered in 25 mCi/m2 every two weeks until the emergence of related grade 2 toxicity. Planar/SPECT ^177^Lu-J591 imaging was performed after six to eight days of the first dose with another optional planar scan after the fourth dose. PSA and circulating tumor cell (CTC) counts were measured before and after treatment along with standard monitoring. Patients received three to six doses (cumulative 75−150 mCi/m2). Two (33%) patients had more than a 30% PSA decline and three (50%) had a CTC count decline.

Myelotoxicity was again the most commonly observed adverse event with two patients (33%) who experienced grade 4 neutropenia (without fever), three (50%) had grade 4 thrombocytopenia (without hemorrhage), and two (33%) required platelet transfusions. Two patients developed worsening cytopenia after their initial recovery from myelotoxicity. Bone marrow biopsies in these two patients later revealed an infiltrative tumor replacing normal marrow elements without myelodysplasia, indicating that the cytopenia was tumor-related and not due to ^177^Lu-J591 toxicity. The targeting of known disease sites was seen on planar imaging in all the patients. The investigators concluded that the hyperfractionation of ^177^Lu-J591 was feasible but failed to demonstrate a significant advantage over the two-dose fractionation regimen.

## Conclusions

Radioimmunotherapy using ^177^Lu-J591 has shown considerable therapeutic efficacy in mCRPC treatment. Anticipated myelosuppression was consistently the most evident toxicity in all J591 therapeutic trials; however, in nearly all cases, myelosuppression was tolerable and reversible. Multiple treatment schedules have been investigated over the years, but to date, dose fractionation into two doses, two weeks apart, appears to be the most promising one. To this end, a phase II registration study of ^177^Lu-J591 (also known as TLX591) is planned and will follow a two-dose fractionation schedule.
